# Nickel-Containing Perovskites, PrNi_0.4_Fe_0.6_O_3–δ_ and PrNi_0.4_Co_0.6_O_3–δ_, as Potential Electrodes for Protonic Ceramic Electrochemical Cells

**DOI:** 10.3390/ma15062166

**Published:** 2022-03-15

**Authors:** Artem P. Tarutin, Anna V. Kasyanova, Gennady K. Vdovin, Julia G. Lyagaeva, Dmitry A. Medvedev

**Affiliations:** 1 Laboratory of Electrochemical Devices Based on Solid Oxide Proton Electrolytes, Institute of High Temperature Electrochemistry, 620990 Ekaterinburg, Russia; 2 Chemical Engineering Institute, Ural Federal University, 620002 Ekaterinburg, Russia; 3 Hydrogen Energy Laboratory, Ural Federal University, 620002 Ekaterinburg, Russia

**Keywords:** PCFCs, PrNiO_3_, EIS, compatibility, electrodes

## Abstract

Protonic ceramic fuel cells (PCFCs) offer a convenient means of converting chemical energy into electricity with high performance and efficiency at low- and intermediate-temperature ranges. However, in order to ensure good life-time stability of PCFCs, it is necessary to ensure rational chemical design in functional materials. Within the present work, we propose new Ni-based perovskite phases of PrNi_0.4_M_0.6_O_3–δ_ (where M = Co, Fe) for potential utilization in protonic ceramic electrochemical cells. Along with their successful synthesis, functional properties of the PrNi_0.4_M_0.6_O_3–δ_ materials, such as chemical compatibility with a number of oxygen-ionic and proton-conducting electrolytes, thermal expansion behavior, electrical conductivity, and electrochemical behavior, were comprehensively studied. According to the obtained data, the Co-containing nickelate exhibits excellent conductivity and polarization behavior; on the other hand, it demonstrates a high reactivity with all studied electrolytes along with elevated thermal expansion coefficients. Conversely, while the iron-based nickelate had superior chemical and thermal compatibility, its transport characteristics were 2–5 times worse. Although, PrNi_0.4_Co_0.6_O_3–δ_ and PrNi_0.4_Fe_0.6_O_3–δ_ represent some disadvantages, this work provides a promising pathway for further improvement of Ni-based perovskite electrodes.

## 1. Introduction

Research into the use of hydrogen as an energy vector continues to increase worldwide as part of rational efforts aimed at balancing the negative impacts of industrial lifestyles with the constant demand for novel energy sources [1,2,3]. In response to increasing risks of global warming and climate change, a number of large-scale hydrogen energy programs have been established over the past few years [4,5,6]. Projects implemented within such programs involve the manipulation of hydrogen and hydrogen-related compounds, including their safe production, storage, transportation, and utilization [7,8]. From this viewpoint, high-temperature electrochemical devices such as solid oxide fuel cells (SOFCs) and electrolysis cells (SOECs), belonging to a broad canvas of hydrogen-related energy approaches, represent one of the most efficient means of energy-conversion for various purposes [9,10,11].

Having been well-studied in terms of fundamental science, conventional SOFCs based on zirconia-based electrolytes are now produced on an industrial scale [12,13,14]. Nevertheless, their relatively high operating temperatures (above 800 °C) result in a certain life-time degradation associated both with interchemical diffusion effects and various microstructural changes [15,16,17]. In this regard, many research efforts have been aimed at decreasing SOFC operating temperatures to intermediate- or low-temperature ranges in order to eliminate such degradation issues [18,19,20]. Here, one of the possible approaches consists in the design of SOFCs based on proton-conducting electrolytes (so-called protonic ceramic fuel cells or PCFCs), which offer desirable performance at reduced operational temperatures due to the high ionic conductivity as a result of proton transportation [21,22,23,24]. Despite the attractiveness of PCFCs, the selection of suitable electrode materials continues to be problematic due to the need to combine superior electrochemical performance with satisfactory compatibility [25,26,27,28,29].

In the present work, we characterize PrNi_0.4_M_0.6_O_3–δ_ (where M = Fe, Co) compounds as possible electrode materials for PCFCs. The justification for selecting these compositions is based on the parent lanthanum-based perovskite phases, i.e., LaNi_1–x_Fe_x_O_3–δ_ (LNF), which demonstrate a good combination between thermal expansion and electrical conductivity [30,31,32,33,34]. In detail, LNF exhibit extremely low thermal expansion coefficients (around (11.5–12.5)·10^−6^ K^−1^ [30]) compared to complex oxides based on simple (Ba_0.5_Sr_0.5_CoO_3–δ_, Ba_0.5_Sr_0.5_Co_0.8_Fe_0.2_O_3–δ_) or double (GdBaCo_2_O5_5+δ_ NdBa_0.5_Sr_0.5_Co_1.5_Fe_0.5_O_5+δ_) cobaltites; the thermal expansion coefficients of the latter are much higher, falling in a range of (15–30)·10^−6^ K^−1^ [32]. Along with desirable thermal functions, the total conductivity of LNF attains 200–1000 S cm^−1^ depending on the iron/nickel ratio [33]; this allows such materials to be utilized as current collectors instead of the Pt electrodes or as an electronic component of composite materials [31,34]. However, electrochemical performance of the LNF electrodes is insufficient [35], since they exhibit predominantly electronic behavior instead of mixed ionic–electronic conduction; this fact can be explained by a low oxygen deficiency variation in LNF (3–δ ≈ 3) that provides a negligible amount of oxygen vacancies and, correspondingly, low oxygen-ionic conductivity. By replacing lanthanum in LaNiO_3_ with a more redox active element (praseodymium), coupled with a further doping with transition elements (iron or cobalt), it is possible to tailor the defect structure and functional properties of the obtained phases for designing compatible and high-performance electrodes for PCFC applications.

## 2. Materials and Methods

### 2.1. Materials Preparation

Materials of PrNi_0.4_Fe_0.6_O_3–δ_ (PNF) and PrNi_0.4_Co_0.6_O_3–δ_ (PNC) compositions were synthesized via the citrate–nitrate method using nitrates of the corresponding metals: Pr(NO_3_)_3_, Ni(NO_3_)_2_, Fe(NO_3_)_3_ and Co(NO_3_)_3_. Stoichiometric amounts of the precursors were dissolved in distilled water, and then citric acid was added to the homogeneous melt. The molar ratio between the total number of metal cations and citric acid was 1:2. During heating to 320 °C, the obtained solution transformed into a transparent gel and then self-ignited. The formed black colored powder materials were subjected to thermal treatment at 1000 °C for 5 h to remove any organic traces. Then, the powders were ground in acetone in a Fritsch Pulverisette 7 planetary mill (Fritsch, Germany) at a frequency of 350 rpm for 1 h. In order to obtain the desired composition, the powders were compacted and calcined at 1200 °C for 5 h. Next, the samples were analogously ground and pressed into pellets using a hydraulic press with a force of 3 tonnes. The relative density of the resultant ceramic materials exceeded 95% of the theoretical density.

To prepare single electrochemical cells, BaCe_0.6_Zr_0.2_Y_0.2_O_3–δ_ (BCZY) and Ce_0.9_Gd_0.1_O_2–δ_ (CGO) electrolyte materials were synthesized by nitrate–citrate auto-combustion technique. Here, nitrates of the corresponding elements served as precursors and citric acid was used as a fuel. The powdered BCZY and CGO materials were synthesized at 1150 °C and 1100 °C for 5 h, respectively. The oxygen-ionic (ZrO_2_)_0.92_(Y_2_O_3_)_0.08_ (YSZ) electrolyte was prepared in the same way, but with two calcination steps (1000 and 1100 °C for 5 h).

### 2.2. Characterization of Materials

The phase composition and crystalline structure of the obtained powders and ceramic samples was studied by X-ray diffraction analysis using a D/MAX-2200 diffractometer (Rigaku Co. Ltd., Japan) in CuK_α_ emission at a wavelength of λ = 1.54056 Å with an angular range of 20° ≤ 2θ ≤ 80°. The Inorganic Crystal Structure Database (ICSD) was used to determine the initial phase composition. Then, in order to specify the structural parameter, the XRD data were analyzed using the Rietveld method [36] via FullProf program software [37].

The cross-sectional morphology and elemental composition of the single electrochemical cells was characterized by the scanning electron microscopy (SEM) analysis using a Phenom ProX electron microscope (Thermo Fisher Scientific, USA). The images of the surfaces of the studied materials were obtained under back electron scattering (BES) regime with an accelerating voltage of 15 kV.

The chemical interaction between PNF and PNC electrode materials with BaCe_0.6_Zr_0.2_Y_0.2_O_3–δ_, Ce_0.9_Gd_0.1_O_2–δ_ or YSZ electrolytes was studied by mixing them in a ratio of 50:50 wt%. The obtained mixtures were calcined at 1100 °C for 10 h. The presence and composition of the products of chemical interaction were determined by XRD analysis.

The thermomechanical behavior of the electrode materials under heating/cooling modes was studied using a Netzsch DIL 402 C dilatometer (Netzsch, Germany). The measurements were performed in air within a temperature range of 50–1000 °C at a constant heating/cooling rate of 3 °C min^−1^. The linear thermal expansion coefficient values were determined on linear regions of the dilatometry curves.

The electrical conductivity of electrode materials was analyzed using the four-probe DC method at temperatures ranging from 20 to 900 °C. The measurements were performed under cooling mode after 50 °C and 1 h isothermal exposure at each step. In order to provide the necessary contact when measuring electrical conductivity, Pt electrodes were used.

### 2.3. Electrochemical Behavior of PrNi_0.4_Fe_0.6_O_3–δ_ and PrNi_0.4_Co_0.6_O_3–δ_ Electrodes

The electrolyte of the BaCe_0.6_Zr_0.2_Y_0.2_O_3–δ_ composition preliminarily annealed at 1430 °C for 5 h served as a basis for the fabrication of symmetrical cells. The PNF/PNC electrode ink was prepared as follows: the corresponding electrode powder was first ground in a Fritsch Pulverisette 7 planetary mill at 550 rpm for 1 h. Then, a mixture of α-terpineol, ethyl cellulose, and dibutyl phthalate was added to the powder to serve as an organic binder; these components were then mixed in ethyl propylene alcohol. After applying the electrodes to the opposite surfaces of the electrolyte via the airbrush coating method, the cell was sintered at 1000 °C for 1 h.

The obtained symmetrical cells were electrochemically characterized using a Amel 2550 high-current potentiostat–galvanostat (Amel, Italy) with a Materials M520 frequency response analyzer (MaterialsM, Italy) in the temperature range of 400–800 °C in humid air (pH_2_O = 0.03 atm) at frequencies ranging from 1 × 10^−2^ to 1 × 10^6^ Hz and a voltage amplitude of 50 mV. The data were obtained in cooling mode. The experimental spectra fitting was performed by means of the equivalent circuit method using ZView software, Scribner Associates Inc. [38].

## 3. Results and Discussion

### 3.1. Phase Structure and Chemical Compatibility

**Figure 1:**
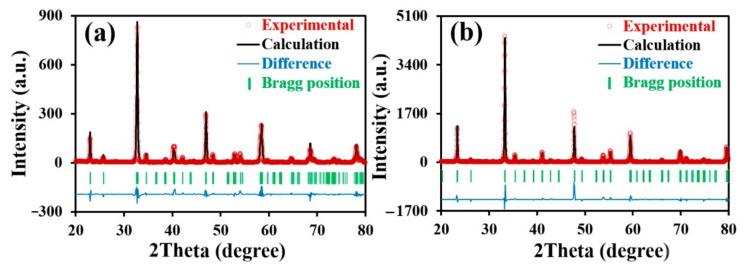
Rietveld refinement results for the PrNi_0.4_Fe_0.6_O_3–δ_ (**a**) and PrNi_0.4_Co_0.6_O_3–δ_ (**b**) at room temperature.

Single-phase materials of the PNF and PNC compositions were obtained using the citrate–nitrate synthesis method. Figure 1 illustrates X-ray diffraction patterns of the studied samples processed by means of the Rietveld refinement. It can be seen that the experimental data agree well with the calculated equivalents. All diffraction peaks are indexable within an orthorhombic perovskite structure described by the Pbnm space group. The refinement parameters of the unit cell for the PNF sample are as follows: *a* = 5.5013 Å, *b* = 7.7309 Å, *c* = 5.4539 Å, and *V* = 231.9 Å^3^. The equivalent parameters for the PNC sample are *a* = 5.3764 Å, *b* = 7.6110 Å, *c* = 5.3885 Å, and *V* = 220.5 Å^3^.

**Figure 2:**
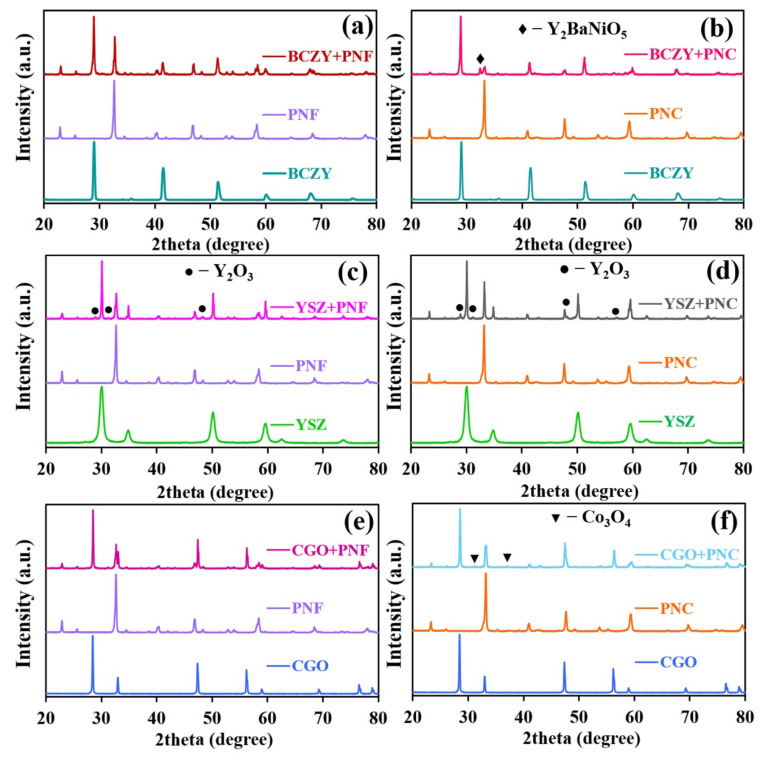
XRD patterns of the PrNi_0.4_Fe_0.6_O_3–δ_ (**a**,**c**,**e**) or PrNi_0.4_Co_0.6_O_3–δ_ (**b**,**d**,**f**) with BCZY (**a**,**b**), YSZ (**c**,**d**), and CGO (**e**,**f**) powders after treatment at 1100 °C for 10 h.

To provide long-term stability, SOFC material components should have good chemical compatibility. Therefore, the chemical compatibility of the PNF and PNC electrode materials was studied with promising BCZY, CGO, and YSZ electrolytes by mixing the corresponding powders and calcinating them at 1100 °C for 10 h. The X-ray diffraction patterns for these calcined mixtures are depicted in Figure 2. 

It was found that the cobalt-containing electrode has low chemical compatibility with all the proposed electrolytes. In detail, the interaction of BCZY with PNC leads to an Y_2_BaNiO_5_ impurity phase, which tends to be localized at the grain boundaries of the electrolyte, leading to considerable ionic transport degradation across the grain boundaries [39,40]. In addition, a significant decrease in the content of the PNC phase in its mixture with the BCZY phase is distinguished. This may be due to a significant decomposition of the initial PNC phase whose cations go to the composition of Y_2_BaNiO_5_ impurity, such as Pr_2_Ba(Ni,Co,Y)O_5_ [41]. In addition, the intensity of reflexes for the PNC phase is also quite small. Therefore, one can conclude that most of the cations were embedded into the BCZY structure, since the Pr [42], Ni [43], and Co [44] cations can be indeed incorporated in the cerium/irconium position. Another possible explanation relies on the assumption that some phases, such as NiO, Co_3_O_4_, Pr_6_O_11_, and Y_2_O_3_, can be localized at an intergrain region. In this case, when they are evenly separated, they become difficult to be detected with XRD.

It is noteworthy that no such interaction occurs in the case of PNF. The chemical reaction occurring between PNC cathodes and YSZ or CGO electrolytes was found to result in the formation of additional Y_2_O_3_ and Co_3_O_4_ impurities along with the basic phases.

The obtained iron-containing nickelate is characterized by its higher chemical stability. Trace amounts of the undesirable yttrium oxide phase are observed in the interaction of the electrolyte with the YSZ-based electrolyte, while other mixtures are relatively stable in terms of forming any impurities, at least at detectable amounts.

### 3.2. Thermal Properties

The absolute oxygen content of the complex oxides was calculated using thermogravimetric analysis (TGA) in pure hydrogen medium when heated to 1000 °C. In order to remove adsorbed gases and moisture, the samples were pre-calcined in air at 1000 °C for 4 h.

It has been assumed that the samples are reduced in hydrogen according to the following reaction:

$${\mathrm{PrNi}}_{0.4}M_{0.6}O_{3-\delta }+\left(1.5-\delta \right)H_{2}\to \frac{1}{2}{\mathrm{Pr}}_{2}O_{3}+0.4\mathrm{Ni}+0.6M+\left(1.5-\delta \right)H_{2}O$$

**Figure 3:**
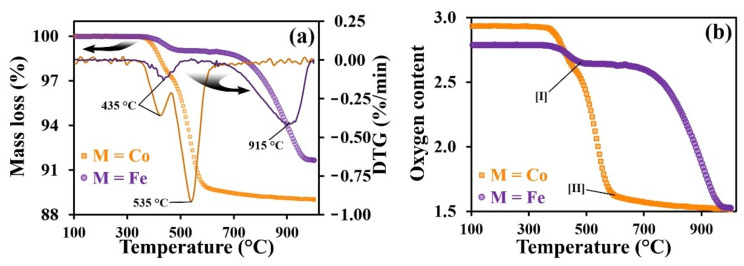
Thermogravimetric data of the PrNi_0.4_Fe_0.6_O_3–δ_ and PrNi_0.4_Co_0.6_O_3–δ_ powders: TGA and DTG data (**a**) and oxygen content in oxides during reduction in H_2_ (**b**).

The released water is removed by gas flow, during which process the samples are observed to undergo weight loss (Figure 3a). On the basis of these changes, the oxygen content of the samples was estimated (Figure 3b): the absolute oxygen content at room temperatures were 2.89 and 2.93 for the Fe- and Co-containing samples, respectively.

On the differential TGA data, there are a number of characteristic peaks (Figure 3a). Similarly, several regions can be distinguished in the temperature dependence of oxygen content (Figure 3b), in which the active processes in the samples are terminated. These are designated as [I] for the Fe-based sample at ~500 °C and [II] for the Co-based sample at ~600 °C. The peak at 435 °C indicates a sharp decrease in weight of both samples; for the PNC, this process is terminated, as shown by point [II]. It is most likely that this weight loss is due to the nickel reduction. The peak at 535 °C was observed for the Co-containing sample, and the oxygen content at point [II] was about 1.67. However, since no further weight loss was detected, this process associated with peak at 565 °C can represent the complete reduction of cobalt-ions to a metallic state. The peak at 915 °C represents the weight loss during the iron reduction. After these processes have been completed, a slow weight reduction to a stable value was observed, indicating the full reduction of the remaining nickel, cobalt, and iron cations.

### 3.3. Thermal Expansion Behavior

**Figure 4:**
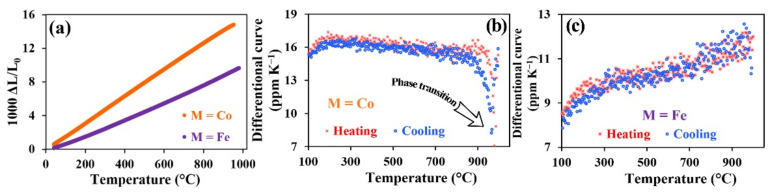
Dilatometry data of the ceramic PrNi_0.4_M_0.6_O_3–δ_ samples: temperature dependence of linear expansion at the cooling (**a**) and differential TEC values (**b**,**c**) obtained at the heating and cooling.

The thermal expansion coefficient (TEC) comprises a basic parameter for SOFC components. Any difference in TEC values between the cathode and electrolyte may result in significant deformations following thermal cycling, which leads to cracking and delamination of components. The TEC values of the studied materials were determined via the dilatometry method. Figure 4a illustrates the temperature dependencies of the relative linear size changes, which were measured at heating from room temperature to 1000 °C.

Figure 4b illustrates differential curves of the dilatometry data for PNC, which clearly show a rapid decrease in TEC values at 950 °C for both cooling and heating. This behavior, according to literature data, is related to a phase transition that occurs due to structural change from orthorhombic to tetragonal lattice symmetry [45]. Apart from that, the lattice expansion, which is observed at elevated temperatures, may be associated with the loss of oxygen in the lattice and consequent formation of oxygen vacancies. The appearance of oxygen vacancies accompanied with the thermal reduction of Co^4+^, Co^3+^, and Ni^3+^ cations to the lower valence states results in the increased inflexion of the thermal expansion curves observed at high temperatures. Since the presence of unstable nickel cations in these oxides is unlikely, it is considered that only cobalt ions are affected by the above-described mechanism [46].

The TEC values calculated using the linear regions of the curves in low-temperature and high-temperature intervals are presented in Table 1.

In the case of PrNi_0.4_Fe_0.6_O_3–δ_, the differential curve (Figure 4c) is convex both before and after the inflexion point (T*). There is a significant difference between the TEC values of iron- and cobalt-containing samples. At temperatures exceeding T*, a chemical expansion effect due to partial iron and nickel reduction processes co-exists along with the dominating thermal expansion. The linear expansion of the samples is associated with the increase in elementary cell volume, which is caused by changes in the ionic radii of iron (from 0.645 Å (HS) for Fe^+3^ or 0.80 Å for Fe^2+^) and nickel (from 0.6 Å (HS) or 0.56 Å (LS) for Ni^+3^ to 0.69 Å for Ni^2+^) cations [47]. The average TEC value for PrNi_0.4_Fe_0.6_O_3–δ_ is equal to 10.2·10^−6^ K^−1^. We note that high-temperature modification of the composition with iron demonstrates a higher TEC value than that obtained from low-temperature modification. A phase transition from orthorhombic to rhombohedral structure may also occur in the temperature interval of the curve break—for example, in LaNi_0.4_Fe_0.6_O_3_ and LaNi_0.2_Fe_0.8_O_3–δ_ [48].

Table 2 illustrates a comparative analysis of the average TEC values of PrNi_0.4_M_0.6_O_3–δ_ and those associated with the most frequently used nickel and praseodymium-based cathode materials [46,49,50,51,52,53]. It was found that the TEC values of the studied materials correspond with the TEC values of other cathode materials. This fact has a favorable effect on the adhesion and thermal compatibility of the studied samples when used as electrolyte materials and makes them attractive for the relevant electrochemical applications. In particular, the TEC values of the studied materials are close to those of the most frequently used electrolyte materials: YSZ (11·10^−6^ K^−1^ [54]), BaCe_0.8_Y_0.2_O_3–δ_ (11.6·10^−6^ K^−1^ [55]), and Ce_0.9_Gd_0.1_O_2–δ_ (12.0·10^−6^ K^−1^ [56]).

Thus, the dilatometric curve form for the studied samples is explained by the mutual influence of the chemical expansion and changes in the phase structure of the materials.

### 3.4. Total Conductivity

**Figure 5:**
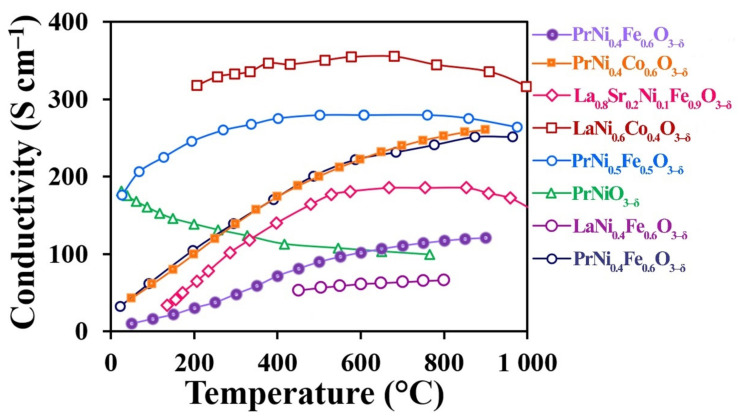
Electrical conductivity of the PrNi_0.4_Fe_0.6_O_3–δ_ and PrNi_0.4_Co_0.6_O_3–δ_ materials compared with electrical conductivities of LaNi_0.6_Co_0.4_O_3‒δ_ [57], PrNi_0.5_Fe_0.5_O_3‒δ_ [58], La_0.8_Sr_0.2_Ni_0.1_Fe_0.9_O_3‒δ_ [53], PrNiO_3‒δ_ [49], LaNi_0.4_Fe_0.6_O_3‒δ_ [59], and PrNi_0.4_Fe_0.6_O_3–δ_ [60].

The high electrical conductivity of the studied materials supports effective electron transfer, which is one of the key factors for their application as SOFC cathodes. The electrical conductivity of sintered samples was studied using the four-probe DC method in the temperature range of 50–900 °C (Figure 5).

The increasing temperature dependence of the electrical conductivity testifies to the semi-conductive character of the studied materials. Their electrical conductivity may be described by the model of hopping conductivity of small radii polarons according to the equation:

(1) $$\sigma =\frac{A}{T}\exp\left(-\frac{E_{a}}{kT}\right),$$

where *A* is the pre-exponential multiplier, S cm^−1^, *T* is the absolute temperature, K, *k* is the Bolstman constant, and *E_a_* is the activation energy. The activation energy values were calculated using the temperature dependence linear regions of the total conductivity. The obtained *E_a_* values are nearly the same for the studied temperatures. Thus, for M = Fe and M = Co, the *E_a_* total conductivity values are 0.27 and 0.32 eV, respectively. Such activation energy values correspond to those of the charge transfer energy barrier according to the small polaron hopping mechanism.

Figure 5 presents the electrical conductivity temperature dependencies for the frequently used electrode materials. It is interesting to note that, according to the literature data, the electrical conductivity of the undoped praseodymium nickelate has a primarily metallic character.

The electrical conductivity values obtained for PrNi_0.4_Fe_0.6_O_3‒δ_ and PrNi_0.4_Co_0.6_O_3‒δ_ at 600 °C are 102 and 222 S cm^−1^, respectively, demonstrating that the studied materials are promising for use in SOFC cathodes.

### 3.5. Electrochemical Characterization

**Figure 6:**
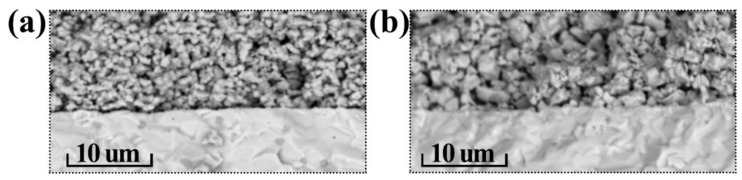
SEM analysis of the PrNi_0.4_M_0.6_O_3–δ_ǀBaCe_0.6_Zr_0.2_Y_0.2_O_3–δ_ interfaces for the prepared symmetrical cells: M = Fe (**a**) and M = Co (**b**).

In order to evaluate the PrNi_0.4_Fe_0.6_O_3‒δ_ and PrNi_0.4_Co_0.6_O_3‒δ_ electrochemical activity, we fabricated symmetrical cells based on the BCZY electrolyte. Due to the microstructure of the electrodes having a significant influence on their electrochemical behavior, we performed SEM analysis of their cross sections prior to characterizing the cells (Figure 6). It is shown that the BCZY electrolyte is dense, and the electrode materials are well-adhered. The formed electrodes have different structural parameters (particle size and porosity). Both factors have a significant impact on the polarization characteristics of these materials.

**Figure 7:**
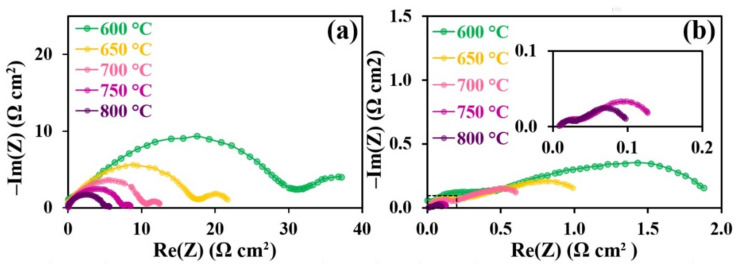
Electrochemical data obtained fothe PNF|BCZY|PNF (**a**) and PNC|BCZY|PNC (**b**) symmetrical cells.

**Figure 8:**
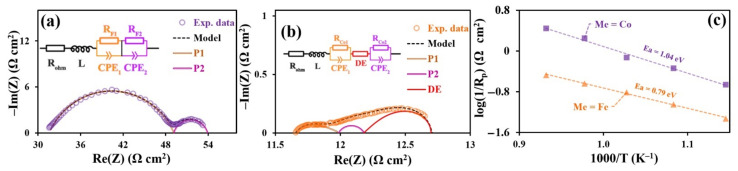
Fitting impedance data of symmetry cells with the PrNi_0.4_Fe_0.6_O_3‒δ_ and PrNi_0.4_Co_0.6_O_3‒δ_ electrodes: used models for fitting impedance data of cells with Fe (**a**) and Co-contended (**b**) air electrodes and temperature dependencies of polarization resistance these cells (**c**).

**Figure 9:**
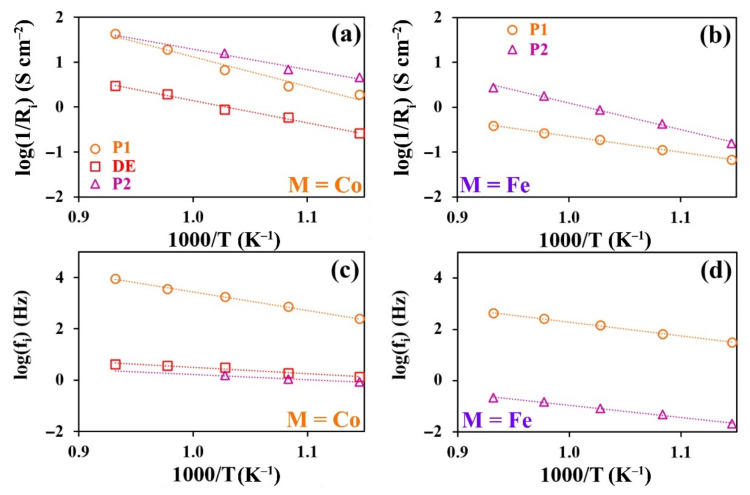
Partial values of the polarization resistances (**a**,**b**) and frequencies (**c**,**d**), determined by fitting of impedance spectra, corresponding to the electrode processes in symmetrical cells for PNC|BCZY|PNC (**a**,**c**) and PNF|BCZY|PNF (**b**,**d**).

The obtained electrochemical impedance spectroscopy (EIS) spectra are illustrated in Figure 7. There is a noticeable difference between the impedance spectra profiles. That is why different electrochemical models with equivalent schemes were used to process the obtained impedance spectra for Me = Fe and Co. The resistor model imitating the ohmic electrolyte resistance, and *L* induction, imitating the frequency reaction of the measurement cell current leads, were used for both samples. The impedance of the Fe-containing sample was fitted using two subsequent R-CPE chains. Regarding the Co-containing sample, a distributed element (DE) applied in the Addler model was used along with two downstream R-CPE chains. The Havriliak–Negami DE impedance is described by the following equation:

(2) $$Z_{\mathrm{DE}}\left(\omega \right)=\frac{R_{\mathrm{chem}}}{{\left(1+{\left(i{\omega \tau }_{\mathrm{chem}}\right)}^{\alpha }\right)}^{\varphi }\;}.\;$$

where $R_{\mathrm{chem}}$. is the resistance of the system associated with the difficulty of current flow during the electrochemical reaction in the porous electrode, and $\tau_{\mathrm{chem}}$ can be regarded as the time constant of the element. At the limiting value of α=1, $\tau_{\mathrm{chem}}$ is equivalent to the cell capacity. In the used model, α→1 and φ~0.5, which brings the applied DE closer to the Gehrischer element. Figure 8 illustrates the models and spectra of the elements. The performed data fitting is used to obtain partial polarization resistance values of symmetrical cells (Figure 9).

For both systems, the P_1_ process is characterized by the capacity in the range from 1 to 7·10^−4^ F·cm^2^, which is characteristic for the flow of H^+^ and O^2−^ ions through the electrode/electrolyte interface [61]. The activation energy values of the polarization resistance calculated from the Arrhenius dependencies were 0.7 and 1.3 eV for Fe- and Co-containing samples, respectively. In the case of the Fe-containing material, the P_1_ process undoubtedly influences the charge transfer through the electrode/electrolyte interface. However, the relatively high activation energy of the polarization conductivity of Co-containing electrodes calls the nature of this process into question. Such activation energy and capacity values were observed for the flow processes of oxygen ions through the electrolyte bulk [62]. Since the process capacities are very similar for both samples, we may conclude that P_1_ is a complex process: the transfer of charge through the electrode/electrolyte interface influences the polarization resistance in the Fe-containing samples, while the polarization process in the Co-containing samples is influenced by the transfer of ions to the electrode volume. The polarization resistance of the symmetrical cell with Fe-containing electrodes significantly exceeds that of the symmetrical cells with Co-containing electrodes. This fact may be associated with the lower conductivity of the Fe-containing nickelate (Figure 5), which is reflected in the processes at the three-phase electrode/electrolyte/air boundary. Another explanation for the difference in P_1_ activation energies between the electrochemical cells with Fe- and Co-containing electrodes is the possible appearance of a small amount of the Y_2_BaNiO_5_ phase (Figure 2b). Given the available data on this phase [63], it is reasonable to conclude that its presence is undesirable and may lead to partial blocking of ion transfer across the interface, leading to an increase in the P_1_ activation energy.

The P_2_ process is characterized by capacities in the range of 0.8–2.0 F·cm^2^ and activation energies of 1.17 and 0.90 eV for the Fe- and Co-containing samples, respectively. The very high observed capacity values are difficult to be associated with the electrochemical nature of the process. In addition, the closeness of the activation energy and capacity values for both cells denotes the common nature of the processes. The nature of the processes may be associated with oxygen adsorption–desorption occurring at the electrode surfaces [64]. The decrease in partial polarization resistance P_2_ observed for the PNC samples, may be caused by the microstructural peculiarities of the material.

Considering the model used, in which DE is analogous to the Gehrischer element, the distributed element process can be related to redox processes in the porous electrodes. The fact that the DE process was not observed for the Fe-containing samples may be explained by the relatively low resistance values as compared to other Fe electrode processes.

Table 3 provides a comparative analysis of the polarization resistance values of symmetrical cells having PrNi_0.4_M_0.6_O_3–δ_ electrodes, where M = Fe, Co and their analogues. Although the comparison is limited by a lack of data for proton-conducting electrolytes among the presented studied materials, the missing values should not differ greatly from those illustrated. It is seen that the polarization resistance values of the studied materials correlated well with their analogues; this is especially characteristic for the co-doped nickelate, which has the smallest polarization resistance value at the studied temperatures.

## 4. Conclusions

The present work provides a comprehensive analysis of the PrNi_0.4_Fe_0.6_O_3–δ_ and PrNi_0.4_Co_0.6_O_3–δ_ complex oxides, which are attractive for application as oxygen electrodes for protonic ceramic fuel cells. These phases were successfully obtained by the citrate-nitrate combustion method followed by high-temperature synthesis. The single-phase powder and ceramic materials were shown to possess certain advantages in their functional properties. On the one hand, Fe-containing samples show good thermal/chemical compatibility with protonic-conducting electrolytes. For example, the average thermal expansion coefficient value of PrNi_0.4_Fe_0.6_O_3–δ_ is considerably lower than that of PrNi_0.4_Co_0.6_O_3–δ_ (10.4 and 15.7 10^−6^·K^−1^, respectively)_._ On the other hand, high conductivity and good electrochemical behavior are observed for the Co-containing nickelate (at 600 °C, 222 S cm^−1^, and 4.6 Ω cm^2^, respectively) compared to its Fe-based counterpart (102 S cm^−1^ and 21.1 Ω cm^2^, respectively). However, both studied materials also demonstrated certain disadvantages, which require further study for improvement of these functions.

## Figures and Tables

**Table 1:** Low-temperature, high-temperature, and average TEC values of PrNi_0.4_M_0.6_O_3–δ_ determined within various temperature ranges and regimes.

Composition	Heating				Cooling			
	T*	100–T*	T*–1000	100–1000	T*	100–T*	T*–1000	100–1000
M = Co	580	16.5	15.5	16.0	550	16.1	15.2	15.7
M = Fe	620	9.9	10.5	10.2	630	9.6	11.1	10.4

**Table 2:** Comparative analysis of the TEC values for Pr- and Ni-containing cathode materials.

Composition	Temperature Interval, °C	TEC·10^6^, K^−1^	References
PrNi_0.4_Fe_0.6_O_3–δ_	100–1000	10.4	This work
PrNi_0.4_Co_0.6_O_3–δ_		15.7	
PrNiO_3–δ_	25–1000	12.7	[49]
PrFe_0.7_Ni_0.3_O_3–δ_	25–900	8.4–11.0	[50]
PrNi_0.4_Fe_0.6_O_3–δ_	400–800	10.5–11.4	[51]
Pr_0.7_Sr_0.3_Fe_0.6_Ni_0.4_O_3–__δ_	25–1000	14.1	[52]
Pr_0.7_Sr_0.3_Fe_0.5_Ni_0.5_O_3–__δ_	25–1000	13.5	[52]
Pr_0.5_Sr_0.5_Co_0.6_Ni_0.4_O_3–__δ_	100–255	15.7	[46]
	400–800	21.6	
	800–1000	27.5	
La_0.9_Sr_0.1_Fe_0.6_Ni_0.4_O_3–δ_	420–700	12.2	[53]
	700–1150	13.1	

**Table 3:** Polarization resistance values of symmetrical cells with PrNiO_3_-based electrodes.

Electrode	Electrolyte	Rp, Ω cm^2^		References
		600 °C	700 °C	
PrNi_0.4_Fe_0.6_O_3–δ_	BCZY	21.1	6.46	This work
PrNi_0.4_Co_0.6_O_3–δ_		4.6	1.3	
PrNiO_3–δ_	Ce_0.9_Gd_0.1_O_2–δ_	0.91	0.13	[49]
Pr_0.7_Sr_0.3_Fe_0.6_Ni_0.4_O_3–__δ_		40.9	4.7	[52]
PrFe_0.6_Ni_0.4_O_3–δ_		20.1	2.5	[60]
PrFe_0.5_Ni_0.5_O_3–δ_		17.8	1.4	
PrFe_0.4_Ni_0.6_O_3−δ_		24.6	2.8	
LaFe_0.4_Ni_0.6_O_3−δ_		3.5	0.4	
PrNi_0.6_Co_0.4_O_3–δ_	Ce_0.8_Sm_0.2_O_2–δ_	0.7	0.1	[65]
LaNi_0.6_Co_0.4_O_3–δ_	La_0.8_Sr_0.2_Ga_0.83_Mg_0.17_O_3–__δ_	34.0	2.9	[66]
LaNi_0.6_Fe_0.4_O_3–δ_	La_0.8_Sr_0.2_Ga_0.83_Mg_0.17_O_3–__δ_	27.3	3.2	
LaNi_0.6_Fe_0.4_O_3–δ_	YSZ	373.2	81.9	[67]
LaNi_0.6_Fe_0.4_O_3–δ_	Ce_0.8_Gd_0.2_O_2–δ_	3.3	0.4	[68]
LaNi_0.6_Fe_0.4_O_3–δ_	Ce_0.9_Gd_0.1_O_2–δ_	21.4	2.7	[69]
LaNi_0.4_Co_0.6_O_3–δ_	YSZ	0.1	0.04	[70]
LaCo_0.6_Ni_0.4_O_3–δ_	Ce_0.8_Gd_0.2_O_2–δ_	326.0	45.0	[71]
LaNi_0.6_Fe_0.4_O_3–δ_	Ce_0.8_Sm_0.2_O_2–δ_	7.6	1.2	[72]

## Data Availability

Data is contained within the article.
